# Safety, feasibility, and hemodynamic response of regadenoson for stress perfusion CMR

**DOI:** 10.1007/s10554-023-02877-z

**Published:** 2023-06-24

**Authors:** Javier Muñiz-Sáenz-Diez, Ana Ezponda, Meylin Caballeros, Ana de la Fuente, Juan J. Gavira, Gorka Bastarrika

**Affiliations:** 1 Department of Cardiology, Clínica Universidad de Navarra, Avenida Pío XII, 36, Pamplona, 31007 Spain; 2 Department of Radiology, Clínica Universidad de Navarra, Pamplona, Spain; 3 Department of Radiology, Clínica Universidad de Navarra, Madrid, Spain; 4 Department of Cardiology, Clínica Universidad de Navarra, Madrid, Spain

**Keywords:** Coronary artery disease, Myocardial perfusion, Regadenoson, Perfusion cardiac magnetic resonance, Drug safety

## Abstract

Owing to its pharmacodynamics and posology, the use of regadenoson for stress cardiac magnetic resonance (CMR) has potential advantages over other vasodilators. We sought to evaluate the safety, hemodynamic response and diagnostic performance of regadenoson stress-CMR in routine clinical practice. All regadenoson stress-CMR examinations performed between May 2017 and July 2020 at our institution were retrospectively reviewed. A total of 698 studies were included for the final analysis. A conventional stress/rest protocol was performed using a 1.5T MRI scanner (Magnetom Aera, Siemens Healthineers, Erlangen, Germany). Adverse events, clinical symptoms, and hemodynamic response were assessed. Diagnostic accuracy of the test was evaluated in patients who underwent invasive coronary angiography. Nearly half of patients (48.5%) remained asymptomatic. Most common clinical symptoms included dyspnea (137, 19.6%), chest pain (116, 16.6%) and flushing (44, 6.3%). Two patients (0.28%) could not complete the examination due to severe hypotension or unbearable chest pain. Overall, an increase in heart rate (HR) response (36.2% [IQR: 22.5?50.9]) and a decrease in systolic and diastolic blood pressure (BP) (median systolic BP response of -5% [IQR: -11.5-0.6]; median diastolic BP response of -6.3 mmHg [IQR: -13.4-0]) was observed. Patients with symptoms induced by regadenoson showed higher HR response (40.3%, IQR: 26.4?56.1 vs. 32.4%, IQR: 19-45.6, p < 0.001), whereas a blunted HR response was observed in diabetic (29.6%, IQR: 18.4?42 p < 0.001), obese (31.7%, IQR: 20.7?46.2 p = 0.005) and patients aged 70 years or older (32.9%, IQR: 22.6?43.1 p < 0.001). Overall, regadenoson stress-CMR showed 95.65% (IQ 91.49?99.81) sensitivity, 54.84% (IQ 35.71?73.97) specificity, 86.99% (IQ 82.74?94.68) positive predictive value, and 77.27% (IQ 57.49?97.06) negative predictive value for detecting significant coronary stenosis as compared with invasive coronary angiography. Regadenoson is a well-tolerated vasodilator that can be safely employed for stress perfusion CMR, with high diagnostic performance.

## Introduction

Coronary artery disease (CAD) has a great impact in morbidity and mortality in the long term [1, 2]. Prompt diagnosis allows adequate management of these patients and improved prognosis. According to the most recent guidelines on chronic coronary syndromes, non-invasive detection of CAD with anatomical or functional testing is recommended for diagnosis and risk stratification in patients in whom clinical evaluation alone cannot rule out CAD [2]. In this context, stress perfusion cardiac magnetic resonance (CMR) has shown superior performance compared to other non-invasive tests [2–5].

Stress-CMR examinations are preferably performed under vasodilator drugs, such as adenosine or dipyridamole [6], which non-selectively target adenosine receptors A1, A2a, A2b, and A3 and cause adverse effects that may limit their use in patients at risk [7, 8]. Regadenoson is a more selective adenosine receptor agonist that preferentially binds to the A2a receptor, responsible for coronary vasodilation. Several studies have shown similar vasodilator effect to that of adenosine [9–11], but with fewer adverse events [12–14]. Although there are many data supporting the effectiveness of adenosine and dipyridamole in the context of stress perfusion CMR [6, 15–21], few studies have evaluated the use of regadenoson.

In this study, we sought to address the safety, feasibility, and hemodynamic response of regadenoson in unselected patients who underwent stress perfusion CMR examinations for clinical indication. We also evaluated the diagnostic accuracy of regadenoson stress perfusion CMR in our patient cohort.

## Materials and methods

### Study population

Between May 2017 and July 2020, 705 consecutive patients with known or suspected coronary artery disease underwent regadenoson stress perfusion CMR. Hemodynamically unstable individuals and patients with myocardial infarction within 24 h, glomerular filtration rate (GFR) < 30 mL/min/1.73 m^2^, or contraindications for regadenoson perfusion CMR were excluded. Patients were instructed to avoid methylxanthine containing substances 24 h prior to CMR examination [22, 23]. Baseline clinical characteristics were collected from electronic medical record data of our institution. Signed informed consent was obtained from all patients and the ethics committee for drug research approved the study protocol, which was performed in conformity with Royal decree 957/2020 and Declaration of Helsinki.

### CMR protocol

CMR examinations were carried out on a 1.5 Tesla system (Magnetom Aera, Siemens Healthineers, Erlangen, Germany) using a conventional stress/rest perfusion protocol, including long and short axis steady state free precession (SSFP) cines, first-pass perfusion imaging under stress and rest conditions, and late gadolinium enhancement (LGE). First-pass stress myocardial perfusion was performed 70 s after the intravenous administration of regadenoson (Rapiscan, GE Healthcare AS) at a fixed dose of 0.4 mg (5 ml). The vasodilator effect of the drug was reverted with euphylline (200 mg i.v.) in all patients, regardless of the clinical symptoms immediately after first-pass stress myocardial perfusion images were acquired, which was approximately 150 s after the administration of regadenoson. A total dose of 0,15 mmol/Kg of gadobutrol (Gadovist, Bayer AG, Berlin, Germany) was administered at 4 ml/s [24].

### CMR image analysis

CMR examinations were analyzed with specific software (cmr 42, Circle Cardiovascular Imaging Inc., Calgary, Canada). Endocardial and epicardial contours were traced in the end-diastolic and end-systolic images to calculate left ventricular volumes, function and mass [25]. The myocardial perfusion was visually assessed. Stress-induced perfusion defects were considered ischemic if the decreased signal intensity involved the subendocardium in a coronary artery territory distribution, the signal intensity was normal during rest perfusion, and the defects did not correspond to myocardial infarction on LGE images. Patients with a positive stress perfusion CMR examination were advised to undergo conventional coronary angiography. The final decision on how to proceed was made individually for each patient by the referring physician.

### Assessment of clinical symptoms, adverse events, and hemodynamic response to regadenoson

Throughout the procedure, ECG tracing, blood pressure (BP) and heart rate (HR) were constantly monitored. All patients were systematically questioned about their symptoms before and after the administration of regadenoson and euphylline, and the predominant symptom was registered. Resting symptoms were asked just before regadenoson administration, while possible vasodilator-related symptoms were asked during its administration, immediately before first-pass stress myocardial perfusion imaging, and just before administration of euphylline. Clinical symptoms were also queried five minutes after euphylline administration to confirm that any symptoms caused by the vasodilator were reversed. In addition, adverse effects that could be related to induced stress, such as bronchospasm, arrhythmias, atrioventricular block, ventricular tachycardia, ventricular fibrillation, need for hospital admission, myocardial infarction or death were collected.

Hemodynamic response to regadenoson was determined by measuring changes in BP and HR under stress and rest conditions (HR response= [(stress HR– rest HR)/rest HR]*100; BP response= ([stress BP – rest BP]/rest BP)*100) [26]. Rest HR and BP data were collected before regadenoson administration. During stress, HR and BP data were registered before contrast administration, immediately after perfusion imaging acquisition and before euphylline injection, and 5 min after euphylline administration. Stress HR was defined as the highest HR during stress perfusion, whereas stress BP was defined as BP taken just after the actual perfusion scan and before the administration of euphylline.

### Diagnostic performance

To establish the diagnostic performance of stress-CMR, sensitivity, specificity, positive and negative predictive values and accuracy were assessed in those patients who underwent invasive coronary angiography in less than one month since the CMR examination. Significant coronary artery obstruction was considered if the fractional flow reserve (FFR) value was < 0.80 or if direct stenting was performed.

### Statistical analysis

Continuous data are described as mean ± standard deviation or as median [interquartile range (IQR)] and compared with the independent sample t-test or using the Mann–Whitney *U* test, as appropriate. Categorical variables are shown as percentages and compared with the Chi-square test. Sensitivity, specificity, positive and negative predictive values and accuracy of regadenoson stress perfusion CMR with respect to conventional coronary angiography were calculated. The statistical analysis was performed using SPSS (version 23.0 / SPSS Inc., Chicago, IL) and a p value < 0.05 was considered statistically significant.

## Results

### Study population

**Fig. 1:**
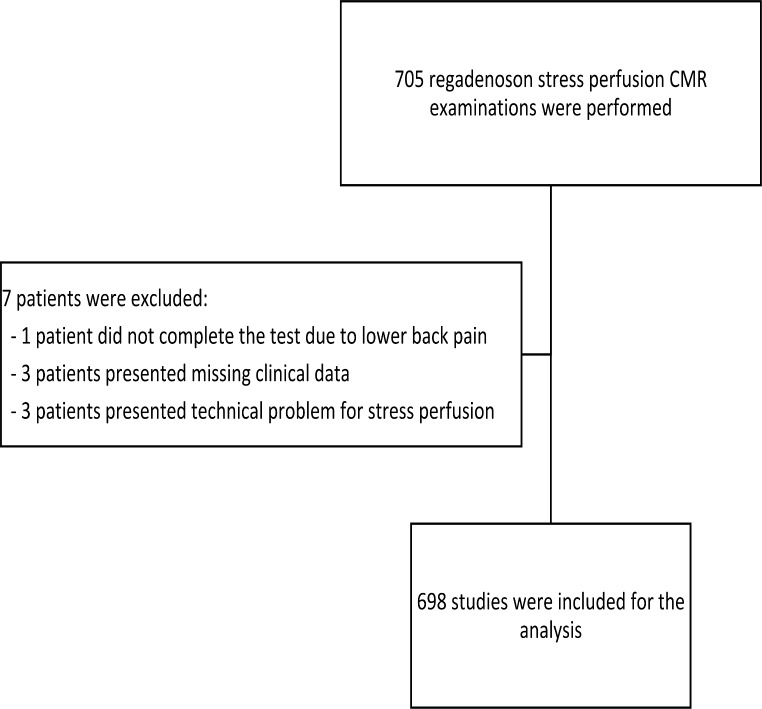
Flowchart of included patients

Seven of the initially included 705 patients were excluded due to technical problems for stress perfusion in three patients, missing clinical data in three patients, and lower back pain that impeded to complete CMR examination in one patient (Fig. 1).

Therefore, a total of 698 patients were considered for the final analysis. The population consisted mainly of men (75.5%) with a median age of 66 years (IQR: 56–73) and a median body mass index (BMI) of 27.1 Kg/m^2^ (IQR: 24.5–30). Most individuals were in sinus rhythm (74.5%). Patient demographics, clinical characteristics, and indications for stress-CMR are shown in Table 1.

**Table 1:** Patient demographics, clinical characteristics, and indications for regadenoson stress perfusion CMR.

Demographics	Patients (n = 698)
Age (years)	66 (56–73)
Elderly (≥ 70 years) (%)	262 (37.5)
Gender (female/male) (%)	171 (24.5) / 527 (75.5)
Height (m)	1.70 (1.64–1.75)
Weigh (kg)	78 (70-88.9)
BMI (kg/m^2^)	27.1 (24.5–30)
BSA (m^2^)	1.91 (1.78–2.06)
Sinus rhythm (%)	520 (74.5)
**Cardiovascular risk factors**	
Smoker/former smoker (%)	407 (58.3)
Hypertension (%)	423 (60.6)
Dyslipidemia (%)	434 (62.2)
Diabetes mellitus (%)	176 (25.2)
Obesity (BMI ≥ 30 Kg/m^2^) (%)	177 (25.4)
Family history of CAD (%)	201 (28.8)
Prior coronary bypass (%)	34 (4.9)
Prior coronary stent (%)	207 (29.7)
Chronic kidney disease (%)	31(4.4)
COPD/Asthma (%)	95 (13.6)
OSAHS (%)	71 (10.2)
**Baseline medication**	
ACEi/ARBs (%)	345 (49.4)
Aspirin (%)	318 (45.6)
Antiplatelet P2Y12 (%)	122 (17.5)
Oral anticoagulation (%)	110 (15.8)
Beta-blockers (%)	282 (40.4)
**Clinical indication for stress-CMR**	
Previous revascularization (%)	231 (33.1)
Suspected cardiomyopathy (%)	164 (23.4)
Angina or equivalent (%)	136 (19.5)
Previous CCTA or exercise ECG (%)	57 (8.2)
High risk profile (%)	38 (5.4)
Ventricular tachycardia (%)	36 (5.2)
Heart transplant (%)	36 (5.2)

Note. Data are presented as median (interquartile range, IQR) or as percentages (%). m: meter; kg: kilogram; BMI: body mass index; BSA: body surface area; CAD: coronary artery disease; COPD: chronic obstructive pulmonary disease; OSAHS: obstructive sleep apnea/hypopnea syndrome; ACEi: angiotensin-converting enzyme inhibitors; ARBs: Angiotensin II receptor blockers; CCTA: coronary computed tomography angiography; ECG: electrocardiogram.

### Clinical symptoms and adverse events

**Fig. 2:**
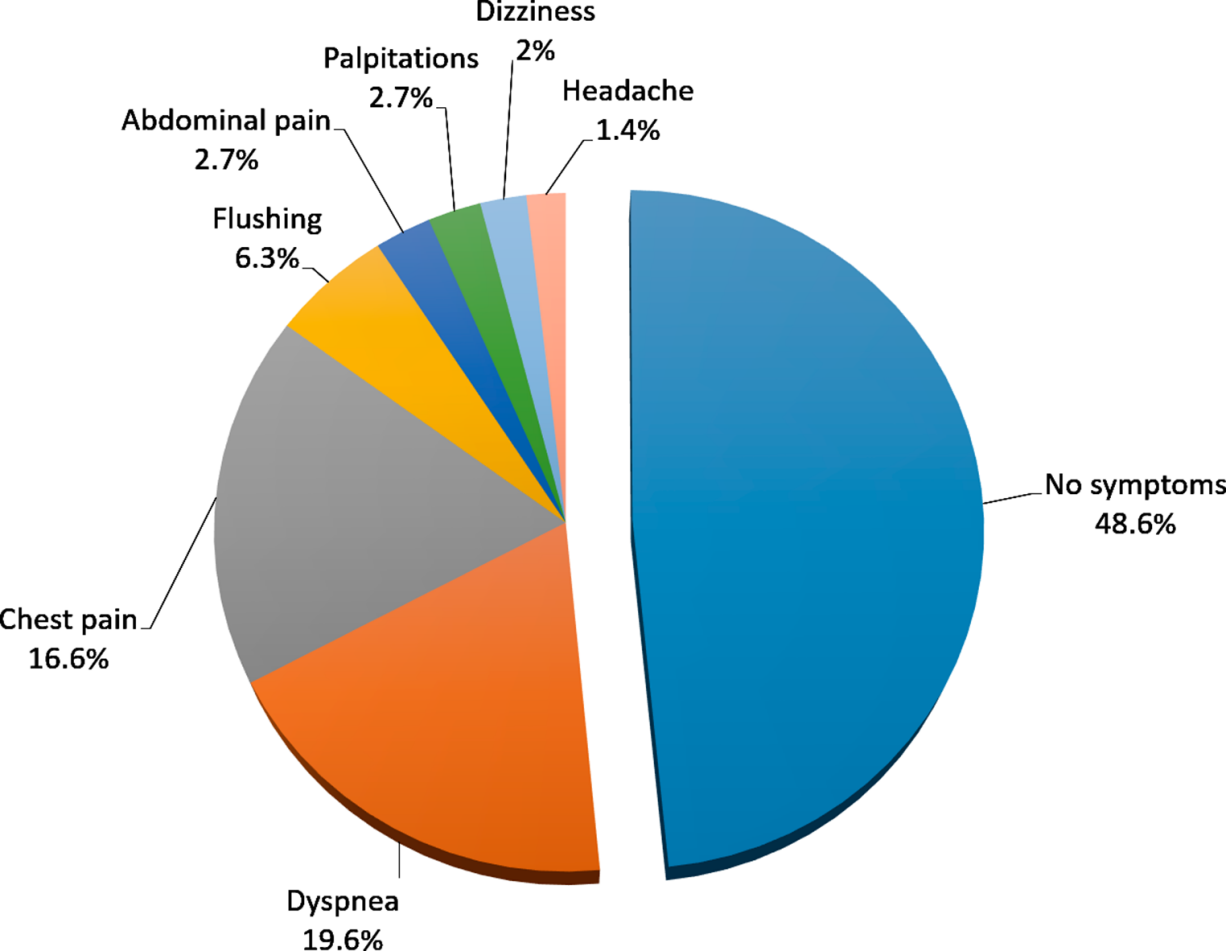
Frequency of symptoms induced by regadenoson

Nearly half of patients (48.5%) remained completely asymptomatic after regadenoson administration. Most common clinical symptoms were dyspnea (19.6%) and chest pain (16.6%). These symptoms were mild, transient, and well tolerated (Fig. 2).

Adverse events included transient stress-induced ectopies (1.7%), transient atrioventricular block (0.28%), bigeminy (0.14%), a limited episode of chest pain that required nitroglycerine administration (0.14%), and contrast extravasation (0.14%). Severe adverse events that prevented completion of the exam were rare (0.28%). One patient suffered regadenoson-induced symptomatic hypotension that required intravenous fluid therapy, whereas another patient referred chest pain that was treated conservatively. No cases of regadenoson-induced atrial fibrillation, ventricular tachycardia, ventricular fibrillation, need for hospital admission, myocardial infarction or death were observed (Table 2).

**Table 2:** Adverse events associated with regadenoson

Adverse event	n = 698
Transient high grade AV block	2 (0.28%)
Bigeminy	1 (0.14%)
Induced atrial fibrillation	0
Ventricular ectopy	12 (1.7%)
VT/VF	0
Bronchospasm	0
Hospitalization	0
Symptomatic hypotension	1 (0.14%)
Chest pain requiring treatment	2 (0.28%)
Contrast extravasation	1 (0.14%)
Myocardial infarction	0
Death	0
Total	17 (2.68%)

Note. Data as presented as number (%). AV: atrioventricular, VT: ventricular tachycardia, VF: ventricular fibrillation

### Hemodynamic response to regadenoson

Resting median HR was 63 bpm, (IQR: 37–127), median systolic BP was 152 mmHg (IQR: 135.75-165.25) and median diastolic BP was 76 mmHg (IQR: 68–83 mmHg). During the stress, the median HR was 87 bpm (IQR: 78–99), the median systolic BP was 144 mmHg (IQR: 128–157) and the median diastolic BP was 71 mmHg (IQR: 63–79 mmHg). Regadenoson induced an increase in HR response (median 36.2%, IQR: 22.5–50.9), and a decrease in systolic and diastolic BP (median systolic BP response of -5%, IQR: -11.5-0.6; median diastolic BP response of -6.3 mmHg, IQR: -13.4-0).

Patients with symptoms induced by regadenoson showed higher HR response (median 40.3%, IQR: 26.4–56.1) compared to individuals that remained asymptomatic (median 32.4%, IQR: 19-45.6) (p < 0.001). Conversely, blunted HR response was observed in obese (median 31.7%, IQR: 20.7–46.2 vs. median 37.2%, IQR: 23-53.7 in non-obese, p = 0.005), diabetic (median 29.6%, IQR: 18.4–42 vs. median 38.1%, IQR: 24.2–54.4 in non-diabetic, p < 0.001) and patients aged 70 years or older (median 32.9%, IQR: 22.6–43.1 in elderly vs. median 41.8%,IQR: 30.3–53.2 in non-elderly, p < 0.001). No statistically significant differences were observed in BP response. Patients under chronic treatment with beta-blockers did not show differences in the hemodynamic response compared with those untreated (Table 3).

**Table 3:** Hemodynamic response to regadenoson across subgroups of patients

	Symptoms			Obesity			Diabetes			Age 70 years or older			Betablockers		
	Yes	No	p value	Yes	No	p value	Yes	No	p value	Yes	No	p value	Yes	No	p value
**HRR**	40.3% (26.4–56.1)	32.4% (19-45.6)	< 0.001	31.7% (20.7–46.2)	37.2% (23-53.7)	0.005	29.6% (18.4–42)	38.1% (24.2–54.4)	< 0.001	32.9% (22.6–43.1)	41.8% (30.3–53.2)	< 0.001	37.8% (26.7–48)	38.9%: (27.5–50.5)	0.49
**SBPR**	-5% (-10.7 -1.3)	-5.4% (-12.7 -0)	0.22	-4.3% (-10.2 -1.3)	-5.2% (-12-0)	0.15	-5% (-12.9 -0)	-5.1% ( -11.5 -0.7)	0.24	6.1% (-12.7 -0.4)	-4.6% (-10.1 -1.2)	0.08	-5.2% (-10.6 -0.2)	-4.9% (-11.1 -1.3)	p = 0.71
**DBPR**	-6.8% (-13.6 -0)	-5.7% (-12.9 -1.2)	0.21	-4.7% (-12.1-0)	-6.6% ( -14.3 -1.1)	0.23	-6.6% ( -14.3 -1.3)	-6.2% ( -12.7 -0)	0.57	-6.6% ( -14.7 -1.4)	-5.2% ( -11.2 -1)	0.24	-4.9% (-13.9 -2)	-6.2%, IQR: -13-0.7	p = 0.21

Note. Data are presented as median (interquartile range, IQR). HRR: heart rate response, SBPR: systolic blood pressure response, DBPR: diastolic blood pressure response.

### CMR findings

CMR findings are shown in Table 4. Mean LV ejection fraction was 66.3 ± 12.7%, mean indexed end-diastolic volume was 72.3 ± 23.3 ml/m2, and mean indexed end-systolic volume was 26.3 ± 19.3 ml/m2. More than half of individuals (54.7%) had normal left ventricular morphology. Almost two thirds of patients showed late gadolinium enhancement (30% with an ischemic pattern and 32% with a non-ischemic pattern).

**Table 4:** CMR results

LV EF, % (sd)	66.3 ± 12.7
LV ESVI, ml/m2 (sd)	26.3 ± 19.3
LV EDVI, ml/m2 (sd)	72.3 ± 23.3
LV mass index, g/m2 (sd)	70 ± 17
RV EF, % (sd)	63.1 ± 8.8
RV ESVI, ml/m2 (sd)	71.1 ± 19
RV EDVI, ml/m2 (sd)	26.2 ± 19.3
**Perfusion and fibrosis**	
Positive stress perfusion (%)	199 (28.2)
LGE ischemic pattern (%)	208 (29.5)
LGE non-ischemic pattern n (%)	140 (19.9)
**Left ventricular morphology**	
Normal	382 (54.7%)
Concentric remodeling	109 (15.6%)
Asymmetric hypertrophy	31 (4.4%)
Concentric hypertrophy	81 (11.6%)
Eccentric hypertrophy	44 (6.3%)
Dilated	51 (7.3%)

Note. CMR: cardiac magnetic resonance, LV: left ventricle, RV: right ventricle; ESVI = end systolic volume index, EDVI: end diastolic volume index; LGE = late gadolinium enhancement, EF: Ejection fraction.

### Diagnostic performance

**Fig. 3:**
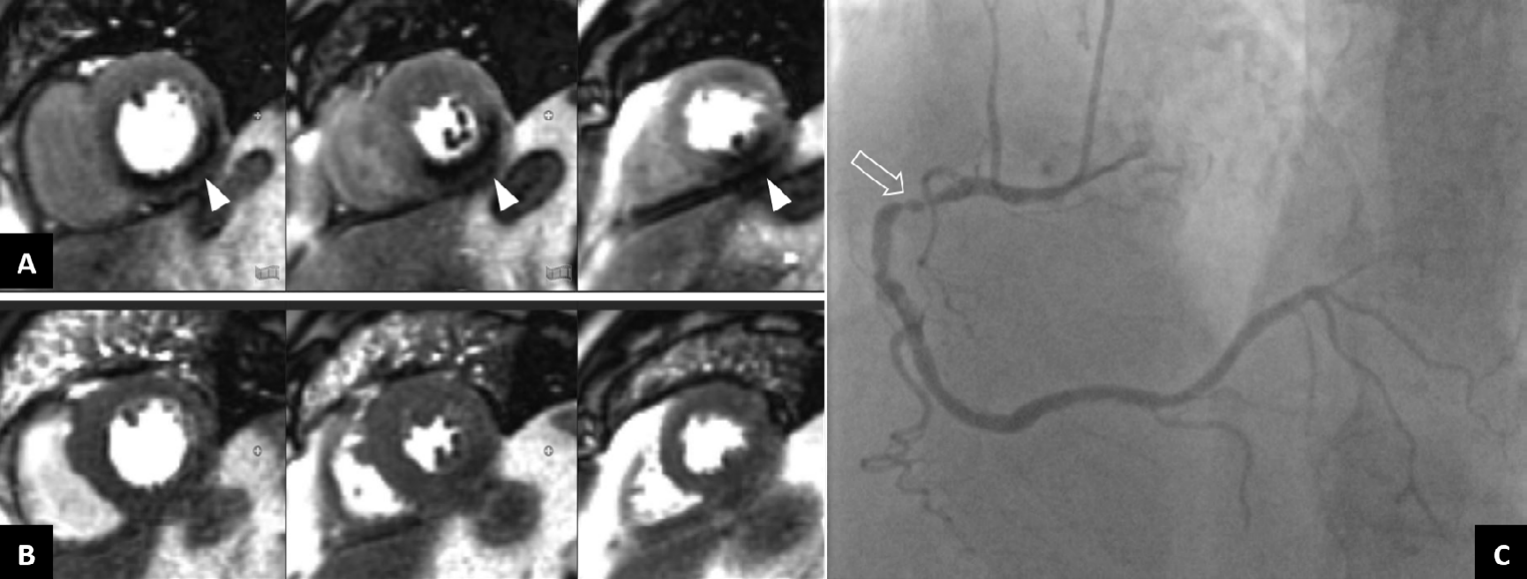
Stress CMR with regadenoson in a 78 year old male with history ofmultiple risk factors (former smoker, hypertension, diabetes mellitus) and percutaneous revascularization of iliofemoral axis stenosis, who presented episodes of chest pain in context of uncontrolled hypertension. (**A**) Stress perfusion. (**B**) Rest perfusion. (**C**) Coronary angiography. The test showed a perfusion defect in the basal, mid and apical inferoposterior segments (arrows in A), with normal perfusion in this segments at rest (image B). This patient underwent invasive coronary angiography that showed severe stenosis in the proximal segment of right coronary artery, and was treated with the implantation of a drug-eluting stent

In our cohort, the regadenoson stress perfusion CMR was positive in 199 patients (Fig. 3). Conventional coronary angiography was performed in 124 with a positive stress-CMR and in 24 patients with a negative stress-CMR examination but with persisting symptoms. The median time to coronary angiography from stress perfusion CMR was 2 days (IQR 1–6, 90th percentile 20.8). Sensitivity for stress CMR was 95.65% (IQ 91.49–99.81) and specificity 54.84% (IQ 35.71–73.97). The positive predictive value was 86.99% (IQ 82.74–94.68) whereas the negative predictive value was 77.27% (IQ 57.49–97.06). There were no statistically significant differences between diabetic and non-diabetic patients in terms of positive stress-CMR (87% vs. 82.4% p = 0.33) nor in the prevalence of significant coronary obstructions (86.7% vs. 73.5% p = 0.06). The diagnostic accuracy was similar (p = 0.07).

## Discussion

The results of this study demonstrate that regadenoson can be used safely in stress CMR examinations. In a routine clinical setting, regadenoson stress perfusion CMR shows high diagnostic performance, comparable to that obtained with other vasodilators.

Stress CMR has many potential advantages over other non-invasive ischemia detection tests. It has a high sensitivity and specificity for diagnosing CAD [3, 5, 19, 27], the technique is the gold standard for evaluating the morphology and function of the heart, does not require ionizing radiation, and the obtained image quality is not influenced by factors such as poor acoustic window. Stress CMR has traditionally been performed with adenosine. The administration of this drug presents, however, some limitations, including the need of an MRI-compatible infusion pump, patient weight based dosage calculation, and the relative contraindications in certain subgroup of patients, such as those with severe respiratory disorders (asthma, chronic obstructive pulmonary disease). Regadenoson may help overcome most of these limitations [12–14, 28, 29].

Several publications have emphasized the safety of regadenoson in nuclear medicine perfusion examinations [8, 30, 31] but studies evaluating the safety profile of regadenoson in CMR are scarce [28]. All the series agree that regadenoson presents fewer complications and better tolerability than adenosine. However, the incidence of symptoms related to the administration of regadenoson varies between the publications. For example, in our study we observed a lower incidence of minor symptoms compared to studies that used nuclear medicine imaging techniques [8, 9] but higher than that reported, for example, by a recent CMR study [29]. Rather than the imaging techniques that were employed, we believe that a more plausible explanation for this finding is the way in which symptoms were reported and collected. We decided to systematically question all patients about any possible regadenoson-induced symptoms at many different points in the study, including before and after euphylline administration, and any side effects related by the patient was thoroughly registered. We also consider that the systematic use of euphylline in all patients may have contributed to better tolerability of the vasodilator. In line with the study by Monmeneu Menadas et al. [29] in our cohort patients with asthma or COPD (n = 96) presented a similar safety profile as the general population, showing no significant adverse events. This observation highlights the safety and tolerability of regadenoson in patients with chronic pulmonary disease. In our cohort, two patients suffered severe events that led to premature test ending. One individual was a 49-year-old male with history of CAD, who referred unbearable chest pain after regadenoson administration. The ECG did not show changes suggesting myocardial ischemia. Prompt euphylline infusion relieved the symptoms. The other patient was an 82-year-old obese male, with systemic arterial hypertension and dyslipidemia under treatment and no history of CAD who was referred for stress CMR for chest pain. Patient’s baseline BP was 133/83 mmHg (HR 55 bpm), and after regadenoson administration it dropped to 65/47 mmHg (HR 98 bpm), presenting as pre-syncope that required rapid euphylline and intravenous fluid administration. No myocardial ischemia was detected in the perfusion exam. In line with other publications, no life-threatening events, hospital admission or death occurred after regadenoson administration.

The significant increase in HR is a distinctive feature that reflects the hemodynamic effect of regadenoson. This vasodilator acts on the sympathetic nervous system through baroreflex-mediated activation and through direct activation of the A2a receptor [28]. In our cohort, we observed blunted HR response in those individuals known to have blunted sympathetic response, including the elderly, obese, and diabetic patients. This finding does not appear to affect test accuracy [26, 30–33], and has been proven to be an independent predictor for poor outcomes in previous studies [33, 34]. Interestingly, patients on chronic beta-blocker treatment did not show a different hemodynamic response to regadenoson, a fact that reassures the performance of stress CMR in the outpatient setting, where medication restriction may not be easy.

All patients received euphylline after stress perfusion despite their clinical symptoms to minimize drug side effects and to reverse regadenoson-induced hyperemia [35]. Being the half-life of regadenoson relatively long as compared with adenosine, concern about residual myocardial hyperemia during the rest perfusion and its impact on the diagnostic accuracy of stress/rest perfusion CMR protocols has been raised. According to our results, however, this fact does not appear to influence the diagnostic performance of the test. Our diagnostic accuracy values are very similar to those reported for stress perfusion CMR. A meta-analysis that compared different cardiac imaging methods against fractional flow reserve (FFR) as the gold-standard to detect lesion-specific ischemia, showed sensitivity of 90% (95% CI, 75–97) and specificity of 85% (95% CI, 79–89%) for stress perfusion CMR [5].

Our study has several limitations. The sample size is smaller than that included by other groups [28–30] and data were retrospectively collected. However, it highlights the routine real-life clinical experience of using regadenoson in unselected patients and adds reassurance to the safety and feasibility of using this vasodilator as stressor in perfusion CMR examinations. The hemodynamic effect of the drug was assessed based on the HR and BP response, without studying hyperemia at the myocardial level, which would have provided a more objective assessment. This requires the use of specific quantitative CMR perfusion sequences that are under active research. In their work, Vasu et al. observed that adenosine and regadenoson have similar vasodilator potency (2.04 ± 0.34 ml/min/g vs. 2.12 ± 0.27 ml/min/g) [10]. Lastly, the number of patients who underwent conventional coronary angiography to confirm CMR findings was low and may limit the interpretation of the diagnostic efficacy of the test. Our results, however, are in line with those reported in the literature.

In conclusion, regadenoson is a safe and well-tolerated vasodilator drug for stress CMR. Adverse reactions are very few and drug-induced clinical symptoms are mild, transient and well tolerated. The hemodynamic response consists of significant HR increase and mild hypotension, which appear to be blunted in those patients with diminished sympathetic response, such as the elderly, obese and diabetic individuals. The diagnostic performance of regadenoson stress perfusion CMR is very similar to that achieved with other vasodilators. Further research is warranted to evaluate the safety and tolerability of regadenoson and its diagnostic performance in specific subgroup of patients.

## List of Abbreviations

AV: Auriculoventricular
BB: Betablocker
CAD: Coronary artery disease
CMR: Cardiac magnetic resonance
COPD: Chronic obstructive pulmonary disease
DBP: Diastolic blood pressure
EDVI: End diastolic volume index
EF: Ejection fraction
eGFR: Estimated glomerular filtration rate
ESVI: End systolic volume index
HR: Heart rate
LGE: Late gadolinium
LV: Left ventricle
RV: Right ventricle
SBP: Systolic blood pressure
VF: Ventricular fibrillation
VT: Ventricular tachycardia

## References

[1] Sorbets E, Fox KM, Elbez Y, Danchin N, Dorian P, Ferrari R (2020). Long-term outcomes of chronic coronary syndrome worldwide: insights from the international CLARIFY registry. Eur Heart J.

[2] Knuuti J, Wijns W, Chairperson I, Capodanno D, France CF, Denmark EP et al (2020) 2019 ESC Guidelines for the diagnosis and management of chronic coronary syndromes the Task Force for the diagnosis and management of chronic. 407–477. 10.1093/eurheartj/ehz425

[3] Nandalur KR, Dwamena BA, Choudhri AF, Nandalur MR, Carlos RC (2007). Diagnostic performance of stress Cardiac magnetic resonance imaging in the detection of coronary artery disease. A Meta-analysis. J Am Coll Cardiol.

[4] Hamon MM, Fau G, Née G, Ehtisham J, Morello R (2010). Meta-analysis of the diagnostic performance of stress perfusion cardiovascular magnetic resonance for detection of coronary artery disease. J Cardiovasc Magn Reson.

[5] Danad I, Szymonifka J, Twisk JWR, Norgaard BL, Zarins CK, Knaapen P (2017). Diagnostic performance of cardiac imaging methods to diagnose ischaemia-causing coronary artery disease when directly compared with fractional flow reserve as a reference standard: a meta-analysis. Eur Heart J.

[6] Menadas JVM, Lopez-lereu MP, Erill JE, Gonzalez AM, Gonzalez PG (2016) Pharmacological stress cardiovascular magnetic resonance: feasibility and safety in a large multicentre prospective registry. 308–315. 10.1093/ehjci/jev153 10.1093/ehjci/jev153 26108417

[7] García-Baizán A, Millor M, Bartolome P, Ezponda A, Pueyo J, Gavira JJ, Bastarrika G (2018). Adenosine triphosphate (ATP) and adenosine cause similar vasodilator effect in patients undergoing stress perfusion cardiac magnetic resonance imaging. Int J Cardiovasc Imaging.

[8] Iskandrian AE, Bateman TM, Belardinelli L, Blackburn B, Cerqueira MD, Hendel RC et al (2007) Adenosine versus regadenoson comparative evaluation in myocardial perfusion imaging: results of the ADVANCE phase 3 multicenter international trial. 645–658. 10.1016/j.nuclcard.2007.06.114 10.1016/j.nuclcard.2007.06.114 17826318

[9] Mahmarian JJ, Peterson LE, Xu J, Cerqueira MD, Iskandrian AE, Bateman TM et al (2014) Regadenoson provides perfusion results comparable to adenosine in heterogeneous patient populations: a quantitative analysis from the ADVANCE MPI trials. 22(2):248–261. 10.1007/s12350-014-9981-6 10.1007/s12350-014-9981-6 25287737

[10] Vasu S, Bandettini WP, Hsu L, Kellman P, Leung S, Mancini C et al (2013) Regadenoson and adenosine are equivalent vasodilators and are superior than dipyridamole- a study of first pass quantitative perfusion cardiovascular magnetic resonance. 10.1186/1532-429X-15-85. 15;85 10.1186/1532-429X-15-85 PMC3851492 24063278

[11] Bateman TM, Thomas GS, Hendel RC, Moye LA, Olmsted AW (2009). Regadenoson induces comparable left ventricular perfusion defects as Adenosine. JCMG.

[12] Doukky R (2014) Regadenoson use in patients with chronic obstructive pulmonary disease: the state of current knowledge. ;129–37 10.2147/COPD.S56879 PMC3904829 24489466

[13] Prenner BM, Bukofzer S, Int M, Behm S, Feaheny K, Mcnutt BE (2012). Study assessing the safety and tolerability of regadenoson in subjects with asthma or chronic obstructive pulmonary disease. J Nucl Cardiol.

[14] Thomas GS, Tammelin BR, Schiffman GL, Marquez R, Rice DL, Milikien D (2008). Safety of regadenoson, a selective adenosine A2A agonist, in patients with chronic obstructive pulmonary disease: a randomized, double-blind, placebo-controlled trial (RegCOPD trial). J Nucl Cardiol.

[15] Gabriella Vincenti MD, Masci abPGiorgio (2017) MD, PHD, a, b Pierre Monney, MD, a, b Tobias Rutz, MD, a, b Sarah Hugelshofer, MD, a Mirdita Gaxherri, MD, a Olivier Muller, MD, PHD, a Juan F. Iglesias, MD, a Eric Eeckhout, MD, PHD, a Valentina Lorenzoni, B. Stress Perfusion CMR in Patients With Known and Suspected CAD. J A C C C a r d i o v a s c u l a r I m a g i n g. ;10(5). 10.1016/j.jcmg.2017.02.006 10.1016/j.jcmg.2017.02.006 28412420

[16] Lipinski MJ, Mcvey CM, Berger JS, Kramer CM, Salerno M (2013) Prognostic value of stress Cardiac magnetic resonance imaging in patients with known or suspected coronary artery disease a systematic review and Meta-analysis. JACC 62(9). 10.1016/j.jacc.2013.03.080 10.1016/j.jacc.2013.03.080 PMC3863376 23727209

[17] Bernhardt P, Levenson B (2006). Contrast-enhanced adenosine-stress magnetic resonance imaging feasibility and practicability of a protocol for detection or exclusion of ischemic heart disease in an outpatient setting. Clin Res Cardiol.

[18] Khoo JP, Grundy BJ, Steadman CD (2012). Stress cardiovascular MR in routine clinical practice: referral patterns, accuracy, tolerance, safety and incidental findings. Br J Radiol.

[19] Esteban-Fernández A, Bastarrika G, Castanon E, Coma-Canella I, Barba-Cosials J, Jiménez-Martín M (2019). Prognostic role of stress cardiac magnetic resonance in the elderly. Rev Española Cardiol.

[20] Karamitsos TD, Arnold JR, Pegg TJ, Cheng ASH, van Gaal WJ, Francis JM (2009). Tolerance and safety of adenosine stress perfusion cardiovascular magnetic resonance imaging in patients with severe coronary artery disease. Int J Cardiovasc Imaging.

[21] Karamitsos TD, Ntusi NA, Francis JM, Holloway CJ, Myerson SG, Neubauer S (2010). Feasibility and safety of high-dose adenosine perfusion cardiovascular magnetic resonance. J Cardiovasc Magn Reson.

[22] Tejani FH, Thompson RC, Kristy R, Bukofzer S (2014) Effect of caffeine on SPECT myocardial perfusion imaging during regadenoson pharmacologic stress: a prospective, randomized, multicenter study. 979–989. 10.1007/s10554-014-0419-7 10.1007/s10554-014-0419-7 PMC4008779 24737255

[23] Van Dijk R, Kuijpers D, Dijkman TAMKPRM, Van (2017). Effects of caffeine intake prior to stress cardiac magnetic resonance perfusion imaging on regadenoson- versus adenosine- induced hyperemia as measured by T1 mapping. Int J Cardiovasc Imaging.

[24] Bastarrika G, Ezponda A, García Baizan A, Calvo M, Pueyo JC, Gavira JJ (2020). Safety of regadenoson for vasodilation in cardiac MRI stress tests. Radiol.

[25] Hundley WG, Bluemke D, Bogaert JG, Friedrich MG, Higgins CB, Lawson MA et al (2009 Mar) Society for Cardiovascular magnetic resonance guidelines for reporting cardiovascular magnetic resonance examinations. J Cardiovasc Magn Reson 11:5. 10.1186/1532-429X-11-5 10.1186/1532-429X-11-5 PMC2662831 19257889

[26] Hage FG, Heo J, Franks B, Belardinelli L, Blackburn B (2009). Differences in heart rate response to adenosine and regadenoson in patients with and without diabetes mellitus. Am Heart J.

[27] Schwitter J, Wacker CM, Wilke N, Al-Saadi N, Sauer E, Huettle K (2013). MR-IMPACT II: magnetic resonance imaging for myocardial perfusion assessment in coronary artery disease trial: perfusion-cardiac magnetic resonance vs. single-photon emission computed tomography for the detection of coronary artery disease: a comparative. Eur Heart J.

[28] Nguyen KL, Bandettini WP, Shanbhag S, Leung SW, Wilson JR, Arai AE (2014). Safety and tolerability of regadenoson CMR. Eur Heart J Cardiovasc Imaging.

[29] Monmeneu Menadas JV, García Gonzalez MP, Lopez-Lereu MP, Higueras Ortega L, Maceira Gonzalez AM (2022). Safety and tolerability of regadenoson in comparison with adenosine stress cardiovascular magnetic resonance: data from a multicentre prospective registry. Int J Cardiovasc Imaging.

[30] Reyes E, Staehr P, Olmsted A (2011) Effect of body mass index on the efficacy, side effect profile, and plasma concentration of fixed-dose regadenoson for myocardial perfusion imaging. 18:620–627. 10.1007/s12350-011-9377-9 10.1007/s12350-011-9377-9 21553161

[31] Cerqueira MD, Nguyen P, Staehr P, Underwood SR, Iskandrian AE (2008). Effects of Age, gender, obesity, and diabetes on the efficacy and safety of the selective A2A agonist Regadenoson Versus Adenosine in Myocardial Perfusion Imaging. J A C C C a r d i o v a s c u l a r I m a g. i n g.

[32] Dibella EVR, Fluckiger JU, Chen L, Ho T, Pack NA, Matthews B et al (2012) The effect of obesity on regadenoson-induced myocardial hyperemia: a quantitative magnetic resonance imaging study. ;1435–44. doi10.1007/s10554-011-9949-4 10.1007/s10554-011-9949-4 PMC3463785 21968545

[33] Hage FG, Perry G, Heo J, Iskandrian AE (2010). Blunting of the heart rate response to Adenosine and Regadenoson in Relation to Hyperglycemia and the metabolic syndrome. AJC.

[34] Hage FG, Dean P, Iqbal F, Heo J, Iskandrian AE (2011). A blunted heart rate response to regadenoson is an independent prognostic indicator in patients undergoing myocardial perfusion imaging. J Nucl Cardiol.

[35] Bhave NM, Freed BH, Yodwut C, Kolanczyk D, Dill K, Lang RM (2012) Considerations when measuring myocardial perfusion reserve by cardiovascular magnetic resonance using regadenoson. 1–8. 10.1186/1532-429X-14-89 10.1186/1532-429X-14-89 PMC3552720 23272658

